# Mechanical recycling of CFRPs based on thermoplastic acrylic resin with the addition of carbon nanotubes

**DOI:** 10.1038/s41598-024-62594-y

**Published:** 2024-05-21

**Authors:** Szymon Demski, Michał Misiak, Kamil Majchrowicz, Gabriela Komorowska, Adrian Lipkowski, Karolina Stankiewicz, Kamil Dydek, Bartłomiej Waśniewski, Anna Boczkowska, Hermann Ehrlich

**Affiliations:** 1 Faculty of Materials Science and Engineering, Warsaw University of Technology, 141 Wołoska St., 02-507 Warsaw, Poland; 2 Łukasiewicz Research Network, Institute of Aviation, Al. Krakowska 110/114, 02-256 Warsaw, Poland; 3 Present Address: Centre for Advanced Technologies, Adam Mickiewicz University in Poznań, 10 Uniwersytetu Poznańskiego St., 61-614 Poznań, Poland

**Keywords:** Carbon-fibre reinforced polymers (CFRPs), Thermoplastic resin, Mechanical recycling, Electrical properties, Composites, Materials science, Carbon nanotubes and fullerenes

## Abstract

Carbon fibre-reinforced polymers (CFRPs) are commonly used in aviation, automotive and renewable energy markets, which are constantly growing. Increasing the production of composite parts leads to increased waste production and a future increase in end-of-life components. To improve the recyclability of CFRPs, new materials that fit in with the idea of a circular economy should be used as a composite matrix. One such material is a commercially available thermoplastic liquid resin, Elium® (Arkema, France). In this work, the authors investigated how the mechanical recycling process affects the properties of thermoplastic-based carbon fibre composites. CFRPs with neat Elium® resin and resin modified with 0.02 wt.% single-walled carbon nanotubes or 0.02 wt.% multi-walled carbon nanotubes were manufactured using the resin infusion process. Afterwards, prepared laminates were mechanically ground, and a new set of composites was manufactured by thermopressing. The microstructure, mechanical, thermal and electrical properties were investigated for both sets of composites. The results showed that mechanical grinding and thermopressing processes lead to a significant increase in the electrical conductivity of composites. Additionally, a sharp decrease in all mechanical properties was observed.

## Introduction

Carbon Fibre Reinforced Polymers (CFRPs) are non-metallic structural materials consisting of carbon fibres and polymer resin. Due to their high specific strength, high stiffness, low weight and high performance, CFRPs are very commonly used in the aerospace, automotive, marine and electronics industries^1–3^. Consequently, the global demand for CFRPs has been steadily increasing since 2008. In 2021, around 181 kt of composites were produced, and the demand is expected to reach 285 kt in 2025^1^. The fast expansion of the composites market in aviation, automotive and renewable energy industries results in environmental problems in managing waste, such as cut-offs generated during production and end-of-life (EoF) elements^4^.

Currently, around 80% of fibre-reinforced polymers (FRPs) are manufactured with thermoset resins (e.g., epoxy, polyester, vinylester) as a matrix^4^. However, the cross-linked structure of cured thermosets leads to significant difficulties in recycling FRPs. The curing process is irreversible; thus, thermoset-based composites cannot be melted or reprocessed. Due to the high volume of FRP wastes, particularly wind turbine blades and boats, were landfilled or incinerated, which negatively impacted the environment and economy^5,6^. The EU directives on landfill waste (EU 1999/31/EC)^7^ and waste incineration (EU 2000/76/EC regulations)^8^ restricted these methods of waste disposal, due to that EoF solutions for many composite elements were limited^5,9^. Consequently, FRP products must be reused, remanufactured or recycled at the EoF stage to avoid waste disposal. Alternatively, closed-loop recycling can be applied to maintain materials in use and circulation^4^.

Generally, recycling methods can be categorised into three groups: mechanical, thermal and chemical^10^. These methods can be applied to thermoset-based and thermoplastic-based composites. Mechanical recycling involves grinding, shredding, crushing or milling of composite waste. During the process, the size of waste is reduced until a fine powder or smaller fibrous products are obtained. Due to size reduction, the mechanical properties of fibres are lost and cannot be reused as structural components. The recyclates in powdered form (with higher resin content) and coarser form (with higher fibre content) can be used as fillers in injection moulding, in sheet moulding compounds or as a reinforcement in cement^5^. Additionally, recyclate from thermoplastic-based FRPs can be used in the thermopressing process to manufacture short-fibre laminates. Moreover, the capital investment and operational cost of mechanical recycling are lower than for thermal and chemical recycling methods^6^. Thermal recycling is used to remove the matrix of composite waste by decomposition during thermal treatments in processes such as pyrolysis and fluidized-bed pyrolysis. Operating temperatures are between 400 and 1000 °C, depending on the type of resin^4,11^. The main advantage of thermal recycling processes is the recovery of long fibres with high mechanical properties, which can be reused in structural components. However, without proper optimisation of the process parameters, there is an increased risk of damaging the fibres, resulting in surface defects or deterioration of mechanical properties. Thanks to the high efficiency of thermal recycling, moderate capital investment and operational cost, pyrolysis processes have been commercialised and used on an industrial scale^6^. In chemical recycling, the composite waste is treated with proper solvents to remove the polymer matrix from FRP waste through solvolysis or dissolution. The solvolysis process is used for thermoset-based composites. It leads to the breaking of covalent bonds in the matrix. In contrast, dissolution is used for thermoplastic-based composites and refers to the physical process of dissolving polymer chains into a solvent^11^. The chemical recycling method allows for the recovery of matrix and long fibres. Compared to thermal recycling methods, the quality of recovered fibres is better. Despite that, toxic solvents, high investment, and operational costs limit chemical recycling use on an industrial scale^5^.

With the challenging and costly recycling process and limited solutions at the EoF stage of thermoset-based FRPs, the use of thermoplastics as a matrix resin in structural components needs to be considered. Elium®, a thermoplastic acrylic resin, is an object of interest for many researchers. This liquid resin can be processed by manufacturing methods commonly used for thermosets, such as resin infusion^12^ or pultrusion process^13^. Thanks to that, the cost of FRPs production is lower than that of thermoplastics in the form of granulates. Moreover, the literature findings show that Elium-based composites possess mechanical properties comparable to epoxy-based composites but with higher impact resistance^12,14^. Additionally, the low viscosity (100 mPa·s) of Elium® resin dedicated to the infusion process enables modification with electrically conductive fillers such as carbon nanotubes (CNTs) without difficulties with fibre permeation. Elium-based composites can be remoulded in a thermopressing process or recycled by mechanical/chemical methods. Cousins et al.^15^ investigated different recycling methods of glass fibre-Elium® resin composite parts cutted from large-scale wind turbine blades. Four techniques were investigated: grinding, pyrolysis and dissolution in chloroform as recycling methods and thermoforming as a remoulding method. Moreover, the authors estimated the energy cost and analysed the presented methods economically. According to a previous article, Gebhardt et al.^16–18^ proposed a recycling strategy that enables the recovery of undamaged continuous fibres in the form of scrims and up to 81% of the Elium® matrix. Conducted tests proved that Elium®, epoxy, urethane acrylate and vinylester matrixes can be successfully used with recycled fibres without a significant drop in mechanical properties.

Allagui et al.^19^ investigated the recycleability of Elium® resin by grinding and thermocompression process. Researchers examined the tensile properties of resin after three cycles of recycling. The obtained results showed an increase in elastic modulus and density, which is related to the increased crystallinity rate of the polymer caused by repeating the thermocompression process. However, the milling process caused the polymer chains to break at numerous points, decreasing some of the tensile properties of Elium® resin.

Although CFRPs are exceptional construction materials, there are fields that require multifunctional materials, e.g., aviation or wind turbine industries. It should be noted that CFRPs exhibit high electrical conductivity along the fibre axis, but this conductivity is much lower through-plain the material. Therefore, the need for construction material with low weight and high electrical conductivity motivates research into improving the electrical conductivity of CFRPs by different modifications^20,21^. One way to improve the conductivity of CFRP is to mix the polymer resin with metallic powders (copper, silver, nickel) or carbon-based fillers (CNTs, carbon black, graphene). However, it should be noted that the addition of nanofillers increases the viscosity of the resin, which affects manufacturing processes^22^. Thus, thanks to a high aspect ratio, which results in a lower percolation threshold, nanofillers such as CNTs are used to modify resin to increase the electrical conductivity of CFRPs^23–25^. Despite the exceptional mechanical, thermal and electrical properties, the CNTs’ tendency to agglomerate requires different methods to achieve good nanofiller dispersion in resin^26,27^. In literature, it has been proven that resin modified with CNTs can improve the electrical conductivity of epoxy-based or thermoplastic-based nanocomposites^28,29^. Moreover, in previous work, it has been proven that modification of Elium® resin with a low content of SWCNTs improves the electrical conductivity of CFRPs by 4.5–5.6 times^30^.

In this paper, thermoplastic resin Elium® was modified with SWCNTs and MWCNTs to improve the electrical conductivity of Elium-based CFRPs manufactured by resin infusion. Afterwards, CFRPs were recycled by mechanical grinding, and the thermopressing process was used to manufacture composites from the obtained recyclate. With increasing problems in the management of composite waste, modified thermoplastic-based composites could be successfully implemented as multifunctional material with various possibilities of recycling at the EoF stage in aviation or wind energy industries. Thus, this article aims to investigate selected properties of SWCNT and MWCNT-modified Elium-based CFPRs after mechanical recycling. To the authors' knowledge, the topic of recycling CNT-modified Elium-based composites has yet to be investigated.

## Materials and methods

### Materials

For the preparation of nanocomposites and CFRPs modified with CNTs, a novel thermoplastic acrylic resin Elium® 188 O (Arkema, Colombes, France) was used as a polymer matrix. This liquid resin possesses a low viscosity of 100 mPa·s and a density of 1.01 g/cm^−3^ and is suitable for the resin infusion process. Dibenzoyl peroxide of 75% concentration (Acros Organics, Geel, Belgium) was used as a polymerisation initiator. Two types of CNTs were used as a filler in nanocomposites. SWCNTs with trade name Tuball™ (OCSiAl, Leudelange, Luxembourg) with the following properties: average diameter < 2 nm, a length > 1 μm and purity 75%, and MWCNTs with trade name NC7000 (Nanocyl, Sambreville, Belgium) with the following properties: average diameter ~ 9.5 nm, average length ~ 1.5 µm and purity 90%. Afterwards, for CFRPs manufacturing, a unidirectional carbon fabric with universal sizing and an areal weight of 603 g/m^2^ (Saertex, Saerbeck, Germany) was used.

### Nanocomposites and CFRPs manufacturing

Firstly, two mixtures of Elium® resin modified with 0.02 wt.% SWCNTs or 0.02 wt.% MWCNTs were prepared. To disperse carbon nanofillers in polymer matrix, mixtures were prepared by ultrasonication process. The ultrasonication was performed using the VCX1500 (Sonics & Materials, USA) ultrasonic processor with a maximum frequency of 20 kHz for 1 h with an amplitude of 40%. Additionally, the temperature of the mixture was measured during the whole process. After heating up to 45 °C, the process was stopped until the mixture cooled down.

Next, the CFRPs with a stacking sequence of [0]_6_ were manufactured by infusion process. The process was conducted at ambient temperature and under a vacuum pressure of 0.9 bar during the whole process. Composites were demoulded after 24 h and afterwards post-cured at 80 °C for 2 h. Table 1 presents the names of manufactured composites that are further used in this paper.

**Table 1:** Nomenclature for manufactured composites.

Manufacturing method	CNT content (%)	Type of CNT	Composite name
Vacuum infusion process	–	–	IP_R
	0.02	SWCNT	IP_S
	0.02	MWCNT	IP_M
Mechanical recycling/Thermopressing	**–**	–	MR_R
	0.02	SWCNT	MR_S
	0.02	MWCNT	MR_M

### Mechanical recycling

To grind manufactured CFRPs modified with CNTs, a WL-4 18,5 kW shredder (WEIMA, Ilsfeld, Germany) with 28 concave rotary knives with 40 mm cutting edge length was used. The mechanical shredding process was fully autonomous and automated, putting the machine into operation by the shaft's rotational movement and the pusher's reciprocating movement. The shredded composites were placed inside the hopper before the device was started. After the machine was switched on, the composite materials were pushed against the v-rotor, which resulted in obtaining a shredded composite fraction (recyclate for further processing). Additionally, an exit sieve with an aperture diameter of 10 mm was mounted. Fine and coarse fractions of recyclate were manually re-screened in the chute basket, and scraps with geometries larger than desired were re-shredded.

### Thermopressing

The thermopressing process was carried out using a WPP 50E hydraulic press (Unicraft, Hallstadt, Germany). The machine was equipped with a heating mould and a stamp that allowed to form a uniform square moulding of 10 cm × 10 cm under the influence of temperature and applied load. The composite plates were manufactured under 10 MPa pressure for 10 min at 150 °C.

### Measurement methods

The microstructure observations of manufactured composites were performed using a cold field emission Scanning Electron Microscope (FE-SEM) SU8000 (Hitachi, Tokyo, Japan). The samples of 4 × 10 mm dimensions were sanded using papers and polished using two diamond slurries. Afterwards, samples were coated with an Au–Pd electroconductive layer using 2 kV voltage, 15 mA current, and 80 s. The observations of the prepared samples were made at an acceleration voltage of 5 kV.

Thermogravimetric analysis TGA was carried out using a TGA Q500 thermogravimeter (TA Instruments, New Castle, DE, USA), where samples were tested to 600 ℃ at a heating rate of 10 ℃/min under a nitrogen atmosphere. The thermal stability loss (T_2%_) and material degradation start (T_5%_) temperatures were determined from the obtained TGA curves, which are characterised by a 2% and 5% mass loss, respectively. In addition, the residue of the tested material at 600 ℃ was identified.

A dynamic mechanical analysis (DMA) was conducted to determine the viscoelastic properties of fabricated CFRPs. Based on the obtained curves, the glass transition temperature (T_g_) and storage modulus at room temperature (E`@RT) of the tested materials were determined. The test was carried out using the DMA Q800 analyser (TA Instruments, New Castle, DE, USA) in dual cantilever mode according to the ASTM D7028 standard in the range from 0 to 150 °C with a heating rate of 2 °C/min at a frequency of 1 Hz and an amplitude of 20 μm. The test samples were 60 mm long and 10 mm wide.

The electrical conductivity of fabricated CFRPs and composites after recycling was measured through the sample thickness (Z-direction). The test samples with the dimensions of 10 mm × 10 mm were taken from different sections of CFRPs. Five test samples represented each material. The electrical conductivity was measured using the Keithley 6221/2182A device ( Keithley Instruments, Cleveland, OH, USA) equipped with a measuring stand with copper electrodes. The silver paste (CW7100 from Chemtronics®, Kennesaw, GA, USA) was used to ensure better contact between electrodes and a sample.

Three-point bending tests were performed according to the ASTM D7264 standard using a static testing machine, MTS QTest (MTS Systems Corporation, Eden Prairie, MN, USA), equipped with a 10 kN load cell. Standard rectangular test specimens with dimensions of about 4 mm × 13 mm × 80 mm (thickness x width x length) were used. Five test specimens represented each material. The bending tests were conducted at a constant crosshead speed of 1 mm/min and a span length of 64 mm. A loading nose and side supports had a diameter of 6 mm. From the obtained force–deflection curves, the flexural modulus of elasticity (E_f_), flexural strength (σ_fM_) and flexural strain at break (ε_fB_) were determined according to the ASTM D7264 standard procedure.

**Figure 1:**
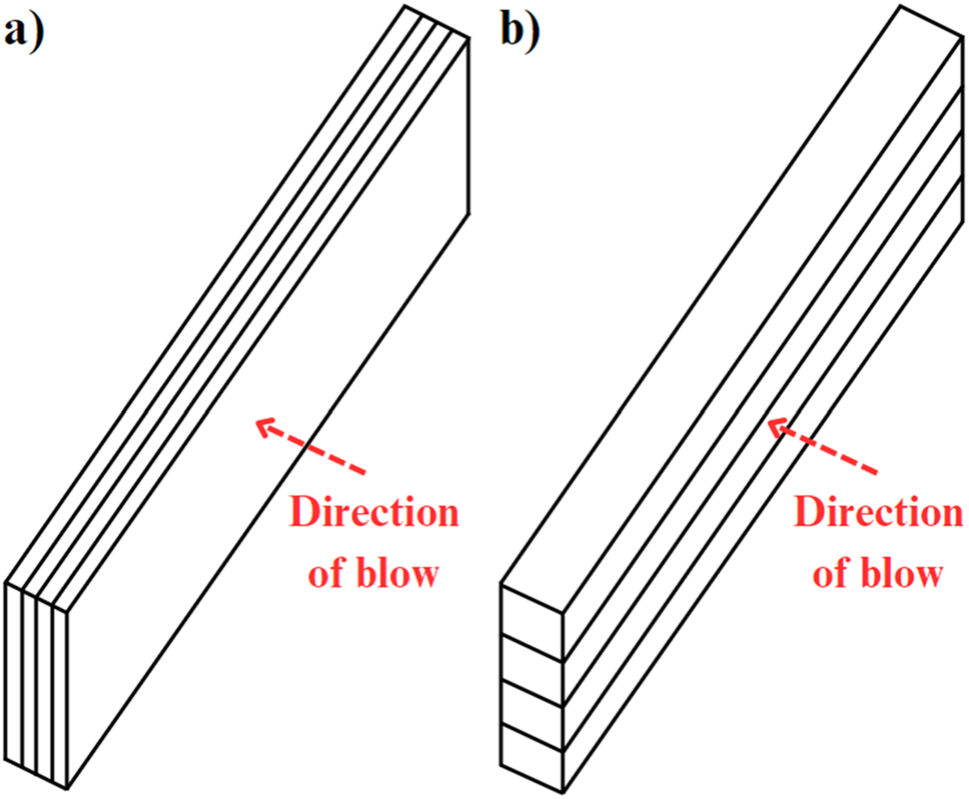
(**a**) Charpy's test for samples with fibre direction perpendicular to to the direction of impact (**b**) Charpy's test for samples with fibre direction parallel to the direction of impact.

The impact strength was determined by Charpy's test using CAEST RESIL 5.5 (impact force of 4 J) for composites with parallel fibres direction to the direction of impact (Fig. 1b) and recycled composites, and Zwick Roell RKP450 (maximum impact force of 300 J) for composites with perpendicular fibres direction to the direction of impact (Fig. 1a). All tests were performed according to the PN-EN ISO 179 standard. Ten unnotched test specimens represented each material. The resin samples were 10 mm × 80 mm in dimensions, and the CFRP samples were 15 mm × 75 mm (width x length) in dimensions. The impact strength was calculated from the absorbed energy obtained at the break and according to the PN-EN ISO 179 standard.

## Results and discussion

### Microstructure observations

**Figure 2:**
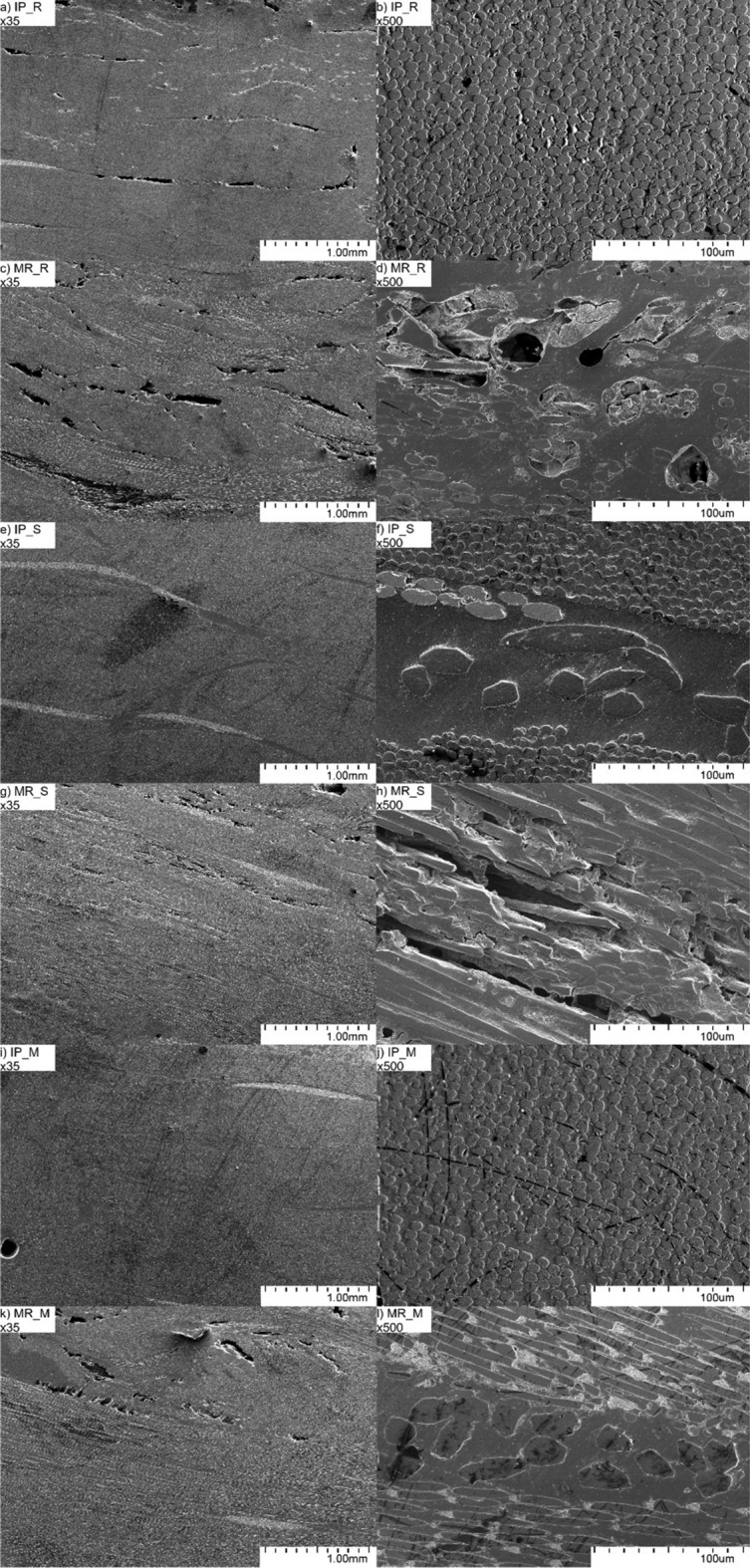
Microstructure of CFRPs manufactured with infusion process: (**a**, **b**) reference (IP_R), (**e**, **f**) modified with 0,02 wt.% SWCNTs (IP_S), (**i**, **j**) modified with 0,02 wt.% MWCNTs (IP_M); and after mechanical recycling: (**c**, **d**) reference (MR_R), (**g**, **h**) modified with 0,02 wt.% SWCNTs (MR_S), k.l) modified with 0,02 wt.% MWCNTs (MR_M).

The microstructure of the manufactured composites before and after mechanical recycling is presented in Fig. 2. Composite samples before the recycling process had a microstructure characteristic for CFRPs consisting of areas with high carbon fibre content and resin-rich regions between carbon fibre layers. All fibres were oriented perpendicularly to the plane of the observed specimen. Additionally, tiny longitudinal pores were observed in resin-rich areas for unmodified sample (IP_R). After the mechanical grinding and thermopressing processes, the microstructure of composites changed significantly. The orientation of carbon fibres was randomised, and the resin-rich areas and areas with high carbon fibre content were difficult to distinguish. Moreover, the random arrangement of fibres and uneven resin-rich area distribution led to the creation of more defects than in samples before recycling. Higher defects amount, shortening and arrangement of carbon fibres cause mechanical properties deterioration. Additionally, it can be observed that composites' quality after recycling differs for each sample. It is connected to the fact that after the grinding process, the obtained recyclate has a random fibre and resin volume fraction. Thus, the thermopressing process should be further analysed to obtain composites of better quality.

### Thermal properties

**Figure 3:**
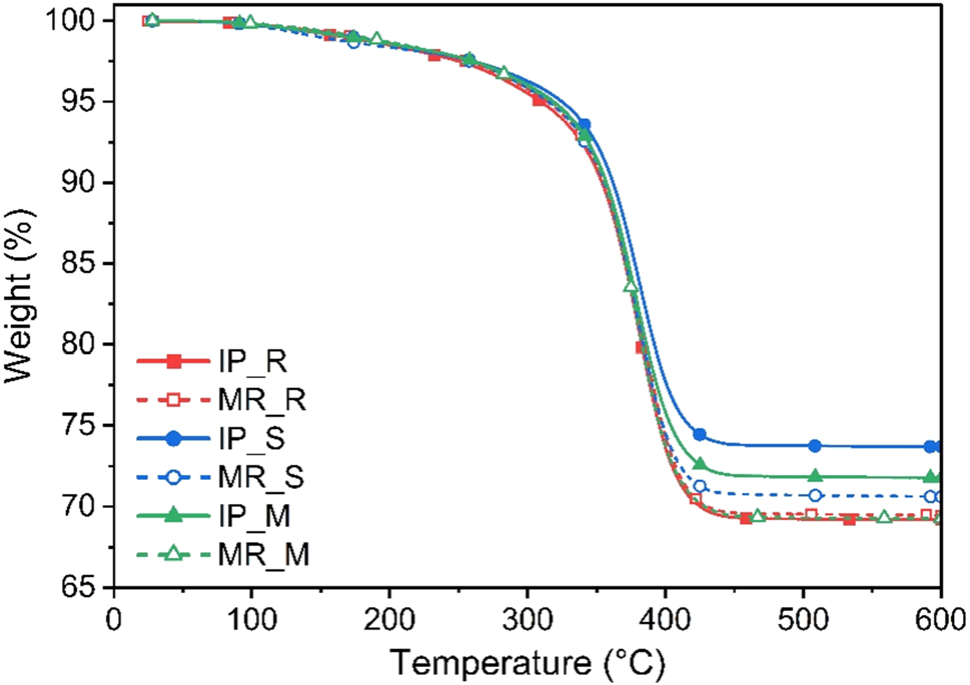
TGA curve of CFRPs before and after the recycling process.

Based on the TGA analysis (Fig. 3 and Table 2), it can be observed that the addition of 0.02 wt.% SWCNTs, as well as MWCNTs, increased the thermal stability and postponed the degradation process of the composite materials before the recycling process, which can be explained by the good dispersion of CNTs in the acrylic matrix. CNTs are known as nanofillers characterised by high thermal conductivity^31^, and their addition caused the formation of an interconnected network and effectively and rapidly distributed thermal energy to the entire composite sample^32^. As a result, local thermal energy accumulation, which leads to faster material decomposition, was prevented. Moreover, the flow of degradation products was hindered due to the presence of CNTs in the acrylic matrix. As a result, the degradation process of the composite material was initiated at a higher temperature^33^. After recycling, the composite material was characterised by fragmentation of CFs randomly distributed in the acrylic matrix. As a result, the effect of the anisotropy of the properties disappeared. Hence, no apparent differences were observed in the thermal stability of the analysed materials. It is noteworthy that the process of recycling and reprocessing did not cause a decrease in the thermal stability of the composite material, which, from the point of view of reuse, is a great advantage. In addition, the grinding and pressing processes had no influence on the change in the weight fraction of CFs, and all the values obtained were around 70% (see Table 2), which confirms the value determined at the laminate manufacturing step.

**Table 2:** The thermal stability loss (T_2%_), material degradation start temperatures (T_5%_) and residue at 600 ℃ of CFRPs before and after the recycling process.

Composite	T_2%_ [℃]	T_5%_ [℃]	Residue at 600 ℃ [%]
IP_R	227	310	69.2
MR_R	232	314	69.5
IP_S	235	325	73.7
MR_S	232	315	70.6
IP_M	238	319	71.8
MR_M	238	317	69.3

**Figure 4:**
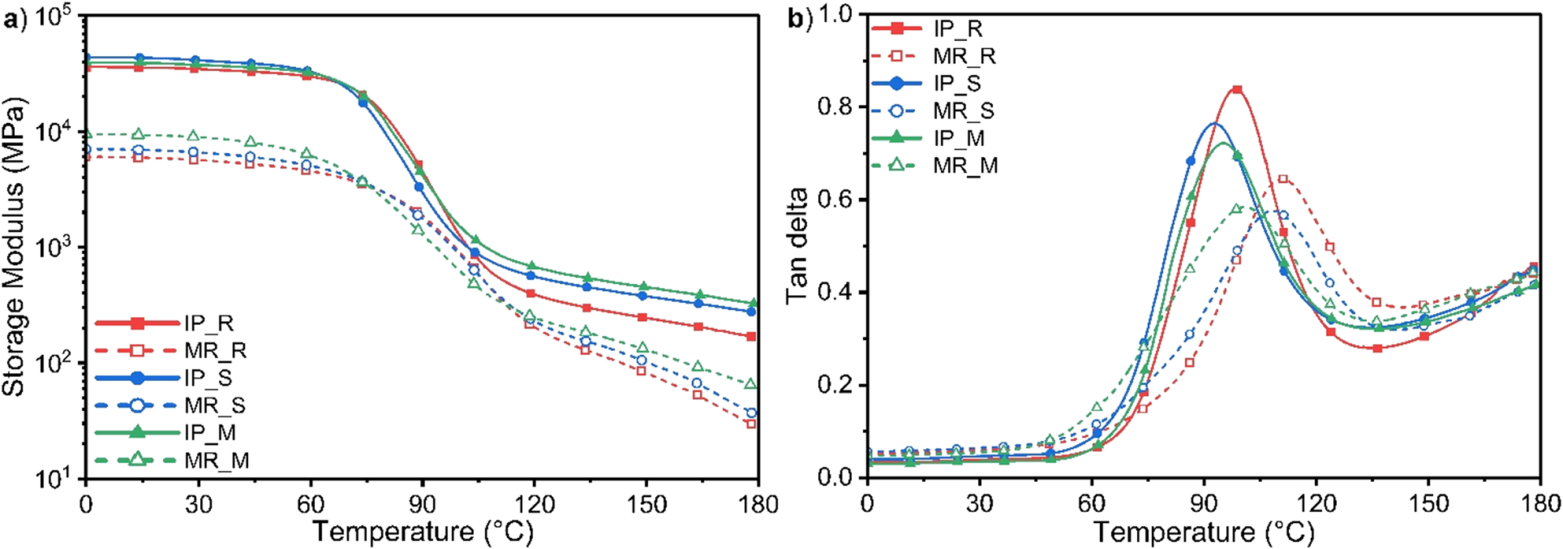
(**a**) Storage Modulus and (**b**) Tan delta of CFRPs before and after the recycling process.

In order to investigate the effects of SWCNTs/MWCNTs addition, milling and pressing on the viscoelastic behaviour and glass transition temperature of the produced CFRPs, DMA analysis was performed. Table 3 and Fig. 4 show the results that were obtained. The glass transition temperature (T_g_) was determined from the TanDelta curve, known as the damping parameter, defined as the ratio of the loss to storage modulus. The peak of the TanDelta curve occurs due to the relaxation of the polymer chains, and T_g_ is related to it^34^.

**Table 3:** Glass transition temperature (T_g_) and Storage Modulus at room temperature (E′_RT_) of CFRPs before and after the recycling process.

Composite	T_g_ [℃]	E′_RT_ [MPa]
IP_R	98	35,102
MR_R	111	5776
IP_S	93	42,057
MR_S	108	6740
IP_M	95	38,096
MR_M	101	9095

For the laminate without CNTs’ addition, a value of T_g_ = 98 ℃ was obtained, and a decrease in T_g_ of 5 ℃ and 3 ℃ was noticed for SWCNTs and MWCNTs, respectively. This could be due to the random distribution of CNTs in the polymer matrix, resulting in the blocking of polymer chains over a high specific surface area of CNTs^35^, which was also observed in^36^. As a result, the addition of both SWCNTs and MWCNTs acts as an inhibitor of the polymerisation reaction. The same phenomenon was observed for nanocomposites after recycling, except that higher values were achieved for all analysed nanocomposites. It could be explained by reprocessing recycled CFRPs and the used thermopressing process parameters. The applied pressing temperature T_press_ = 150 ℃ is the temperature at which the Elium® resin is in a plastic state, and the macromolecules are reconfigured. Moving on to analysing the storage modulus curves, which measure the composite material's elasticity, Table 2 contains the modulus values at room temperature, i.e. RT = 25 ℃. Analysing the E′ values for the laminates before recycling, it is worth noting that the addition of SWCNTs and MWCNTs to the Elium® resin increased the storage modulus. This increase was dictated by the addition of a nanofiller characterised by high mechanical properties^37^, interfacial interactions between the acrylic resin and CNTs^38^, as well as its suitable dispersion. The higher (by 10.4%) E′_RT_ values for IP_S compared to IP_M could be explained by the higher mechanical parameters of SWCNTs^39^. In the case of composites produced after the recycling process, their significantly lower E′_RT_ values were dictated by the form of CFs’ reinforcement. The milling process shortened the CFs and changed their orientation, resulting in a decrease in the composite material's stiffness, which is a challenge for the mechanical recycling of CFRPs^4^.

### Electrical properties

**Figure 5:**
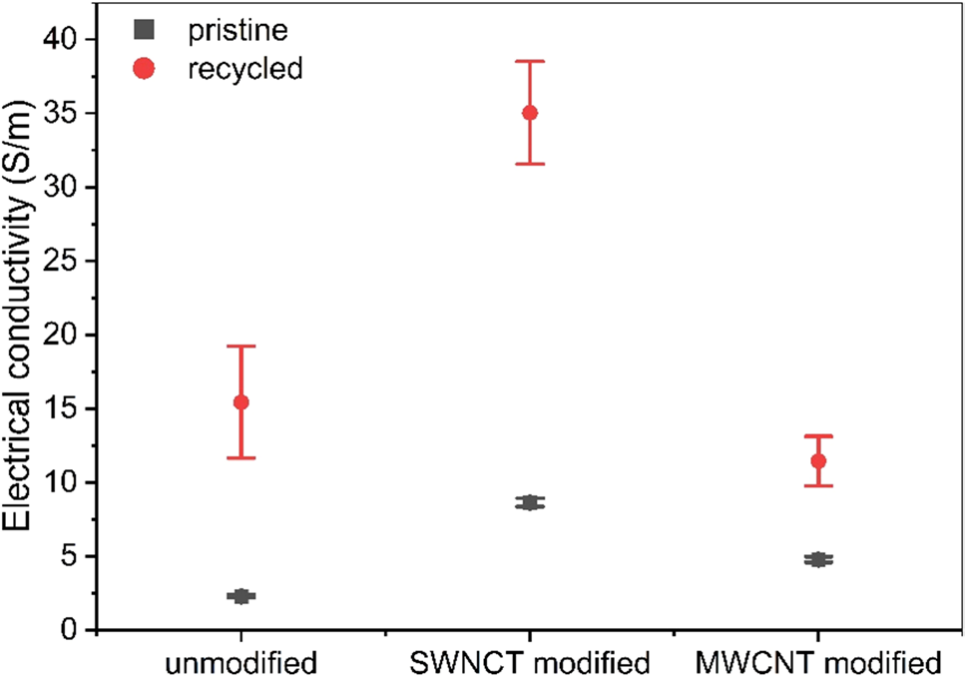
Through-plain electrical conductivity of CFRPs before and after the recycling process.

One disadvantage of CFPRs is their relatively low electrical conductivity, which limits their application possibilities. It is well described in the literature that the addition of carbon nanotubes increases the electrical conductivity of materials^40,41^. Fig. 5 shows the average value of the volume electrical conductivity of the composites before and after the recycling process. The electrical conductivity of the reference composite was 2.3 S/m. The addition of SWCNTs and MWCNTs increased the electrical conductivity by 276% and 108%, respectively. The observed increase in electrical conductivity for modified composites is related to the formation of the conductive paths in resin-reach regions. This phenomenon creates a conductive connection between carbon fibre layers in composite^42^.

Composites, after mechanical recycling, show a significant increase in electrical conductivity. A sevenfold increase in electrical conductivity was recorded for MR_R composites. The MR_S and MR_M composites showed an increase in conductivity of 305% and 139%, respectively, compared to pristine composites. As seen in Section "Microstructure observations" in the composites before recycling, all carbon fibres are arranged perpendicularly to the plane of the sample. Between the layers of carbon fabric is an insulating layer of Elium® resin. In contrast, in the samples after grinding and then thermopressing processes, the carbon fibres are distributed unevenly throughout the composites. Thus, the insulating resin layer is interspersed with conductive carbon fibres, creating multiple conductive paths throughout the composite structure. Therefore, mechanically recycled carbon fibre-reinforced composites show significantly higher electrical conductivity than reference laminates.

It is worth noting that in the case of recycled composites, the addition of SWCNTs also resulted in a noticeable increase in conductivity compared to the unmodified composite. Moreover, composites with high electrical conductivity can find applications as electromagnetic interference shielding materials in the automotive market or as lightning strike protection materials in aviation or wind turbine markets.

### Mechanical properties

The calculated flexural properties are summarised in Table 4. The flexural modulus of elasticity and flexural strength of the IP_R composite (without CNTs) reached values of 95.6 ± 2.2 GPa and 819 ± 15 MPa, respectively. The addition of both SWCNTs, as well as MWCNTs, caused a slight increase of stiffness to a similar level, i.e. the flexural modulus of elasticity was enhanced to 101.4 ± 2.4 GPa and 101.4 ± 1.6 GPa, respectively. In turn, the flexural strength was reduced compared to the reference IP_R composite. It was more evident for IP_S (a reduction of σ_fM_ down to 725 ± 46 MPa) than for IP_M (804 ± 45 MPa). Unfortunately, the recycling process resulted in a drastic diminishment of mechanical properties during bending. After recycling, the flexural modulus of elasticity and flexural strength of the MR_R composite were only 14.3 ± 1.2 GPa and 45.8 ± 10.3 MPa, respectively. In contrary to the results for the non-recycled composites, the highest flexural strength of 62.8 ± 11.9 MPa was noted for MR_M. The lowest flexural strength value was measured for MR_S, i.e. 33.8 ± 11.9 MPa. It is also worth mentioning that all Elium®-based composites after recycling were characterised by a much greater heterogeneity, which resulted in a significant scattering of the obtained bending results. The coefficient of variation (defined as a ratio of the standard deviation to the mean value) of the flexural strength of the recycled composites ranged from 0.19 to 0.35, while the results for Elium-based composites before the recycling process usually differed by about 2–6% (i.e. the coefficient of variation ranged from 0.02 to 0.06).

**Table 4:** Flexural properties of CFRPs before and after the recycling process.

Composite	Flexural modulus of elasticity E_f_ (GPa)	Flexural strength σ_fM_ (MPa)	Flexural strain at break ε_fB_ (%)
IP_R	95.6 ± 2.2	819 ± 15	1.1 ± 0.1
MR_R	14.3 ± 1.2	45.8 ± 10.3	0.6 ± 0.2
IP_S	101.4 ± 2.4	725 ± 46	1.0 ± 0.1
MR_S	10.5 ± 1.3	33.8 ± 11.9	0.9 ± 0.6
IP_M	101.4 ± 1.6	804 ± 45	1.0 ± 0.1
MR_M	12.2 ± 2.4	62.8 ± 11.9	1.2 ± 0.3

Charpy's test was conducted to investigate the effect of MWCNT/SWCNT addition, grinding and thermopressing processes on the impact strength of Elium-based CFRPs. Before the recycling process, tests were carried out for carbon composites in two directions: perpendicular and parallel to the carbon fibres. In the perpendicular direction, the carbon fibres are responsible for load transfer, while in the parallel direction, the Elium® resin mainly takes over this function.

**Figure 6:**
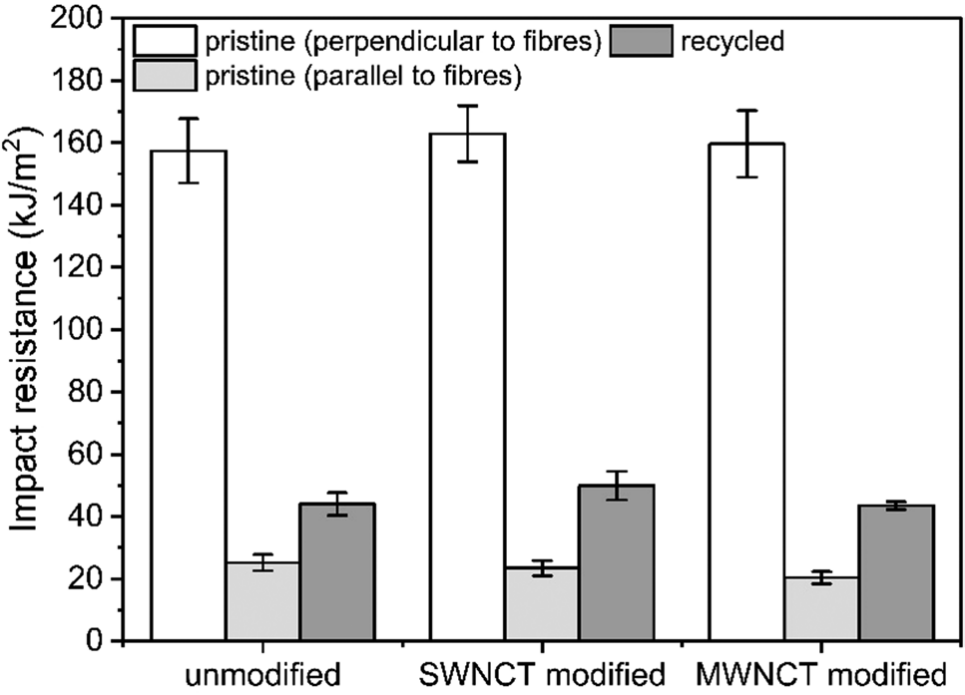
Impact resistance of CFRPs before and after the recycling process.

Analysis of the results indicates that the addition of SWCNTs and MWCNTs in the amount of 0.02 wt.% did not change the impact strength of CFRP (Fig. 6). However, in the literature, one can find a correlation between the increase in the impact strength value with the addition of more CNTs, for example, due to their excellent mechanical properties. The increase in impact strength values with the addition of CNTs is related to factors such as CNTs pullout and bridging^43,44^.

The impact strength for the IP_R composite (unmodified) reached a value of 25.2 ± 2.6 kJ/m^2^ when tested parallel to the carbon fibres and 157.4 ± 10.3 kJ/m2 tested perpendicular to the carbon fibres. When SWCNTs were added, the values reached 23.4 ± 2.5 kJ/m^2^ and 162.9 ± 9.0 kJ/m^2^, respectively. Similar impact resistance values were obtained for composite samples containing MWCNTs: 20.3 ± 2.0 kJ/m^2^ and 159.6 ± 10.7 kJ/m^2^. The mechanical recycling process caused a sharp reduction in mechanical properties (Fig. 6). The impact resistance value of the MR_R was 44.0 ± 3.6 kJ/m^2^. The highest impact value of 49.9 ± 4.6 kJ/m^2^ was recorded for MR_S, and the lowest impact strength value was obtained for MR_M, 43.5 ± 1.3 kJ/m^2^. Grinding and thermopressing processes of the composites increased the number of structural defects compared to the composites before recycling.

## Conclusion

The effects of the mechanical recycling process on the properties of thermoplastic-based CFRPs were investigated in this study. Pristine composites with neat Elium®, SWCNT-modified Elium® and MWCNT-modified Elium® matrix were manufactured by resin infusion. Afterwards, prepared composites were mechanically grinded, and a new set of composites was manufactured by thermopressing process. The mechanical, thermal and electrical properties were investigated for both sets of manufactured samples. Several conclusions can be drawn from conducted studies:

- For composites manufactured by infusion process, the Elium® matrix modification with 0.02 wt.% of SWCNTs or MWCNTs leads to an increase in electrical conductivity (by 276% and 108%, respectively, compared to unmodified composite). Notably, the CNT modification slightly improved the composites' flexural properties and postponed the composites' thermal degradation process. The results showed that matrix modification with low CNT content can enhance the functionality of CFRPs and widen the possibilities for new applications in aviation, automotive and renewable energy markets.
- The Elium-based CFRPs are easily recyclable by mechanical grinding and thermopressing process. Considering that the market for composites is gradually increasing, it is crucial to design composite parts that fit the idea of the circular economy. This can be achieved by substituting conventional epoxy resins with thermoplastic materials such as Elium® resin.
- Mechanical recycling results in shortening the length of carbon fibres. Due to that, the recycled composites’ structure exhibited a heterogeneous microstructure with more defects than the microstructure of pristine CFRPs. These changes in microstructure caused sharp deterioration of composites’ mechanical properties. Moreover, the changed arrangement of carbon fibres leads to the creation of new conductive paths. Thus, the electrical conductivity (an increase of 570%, 305% and 139% for unmodified, SWCNT-modified and MWCNT-modified composites, respectively) was further improved compared to CFRPs manufactured by the infusion process.

In summary, low CNT content modification of Elium® resin results in an electrical conductivity increase without worsening the mechanical properties of CFRPs. Such composites have exceptional mechanical properties and simultaneously offer a new functionality, which meets the demand of the gradually increasing composite industry. Moreover, Elium-based parts are suitable for recycling and can be reused for different applications after EoL. However, it has to be mentioned that mechanical recycling results in a significant decrease in composites’ mechanical properties, which limits the application possibilities.

## Data Availability

The datasets used during the current study are available from the corresponding author upon reasonable request.
